# Non-Alcoholic Fatty Liver Disease in Obese Youth With Insulin Resistance and Type 2 Diabetes

**DOI:** 10.3389/fendo.2021.639548

**Published:** 2021-04-06

**Authors:** Serena Scapaticci, Ebe D’Adamo, Angelika Mohn, Francesco Chiarelli, Cosimo Giannini

**Affiliations:** Department of Pediatrics, University of Chieti, Chieti, Italy

**Keywords:** non-alcoholic fatty liver disease, liver steatosis, steatohepatitis, obesity in children, metabolic syndrome, insulin resistance

## Abstract

Currently, Non-Alcoholic Fatty Liver Disease (NAFLD) is the most prevalent form of chronic liver disease in children and adolescents worldwide. Simultaneously to the epidemic spreading of childhood obesity, the rate of affected young has dramatically increased in the last decades with an estimated prevalence of NAFLD of 3%–10% in pediatric subjects in the world. The continuous improvement in NAFLD knowledge has significantly defined several risk factors associated to the natural history of this complex liver alteration. Among them, Insulin Resistance (IR) is certainly one of the main features. As well, not surprisingly, abnormal glucose tolerance (prediabetes and diabetes) is highly prevalent among children/adolescents with biopsy-proven NAFLD. In addition, other factors such as genetic, ethnicity, gender, age, puberty and lifestyle might affect the development and progression of hepatic alterations. However, available data are still lacking to confirm whether IR is a risk factor or a consequence of hepatic steatosis. There is also evidence that NAFLD is the hepatic manifestation of Metabolic Syndrome (MetS). In fact, NAFLD often coexist with central obesity, impaired glucose tolerance, dyslipidemia, and hypertension, which represent the main features of MetS. In this Review, main aspects of the natural history and risk factors of the disease are summarized in children and adolescents. In addition, the most relevant scientific evidence about the association between NAFLD and metabolic dysregulation, focusing on clinical, pathogenetic, and histological implication will be provided with some focuses on the main treatment options.

## Introduction

Nonalcoholic Fatty Liver Disease (NAFLD) is the most common cause of chronic liver disease in children and adolescents in the developed country resulting from excessive fat accumulation into the liver (1). According to the North American Society of Pediatric Gastroenterology, Hepatology and Nutrition (NASPGHAN), NAFLD is defined as a chronic hepatic steatosis in children not secondary to genetic/metabolic disorders, infections, use of steatogenic medications, ethanol consumption, or malnutrition (2).

The natural history of NAFLD describes different and well characterized degrees of histological hepatic damages. These alterations range from simple nonalcoholic fatty liver accumulation (with a fat content in the hepatocytes higher than 5%) to nonalcoholic steatohepatitis (NASH) characterized by hepatocyte injury and cell death. Finally, the collagen deposition and subsequent vascular remodeling lead to fibrosis, cirrhosis and end-stage liver diseases even occurring in childhood (3).

NAFLD is commonly reported in obese children and adolescents, particularly in those with metabolic syndrome (MetS). Its prevalence in obese youth has shown a significant increase paralleling the epidemic of childhood obesity during the last years. In the United States, one in three children is currently afflicted with overweight or obesity (4) and recent data have reported an increased prevalence of this conditions by 8 to 8.75-fold since 1975 (5). The currency of childhood obesity conducts to high emergence of comorbidities previously described only in adulthood such as type 2 diabetes mellitus (T2DM), hypertension, obstructive sleep apnea, dyslipidemia, and NAFLD (4, 6–10). Many studies have reported a strong relationship between NAFLD and insulin resistance (IR), which is commonly considered to be the shared and main component of the metabolic alterations characterizing MetS. Although several definitions of MetS in children have been provided, data collected from recent reports consider central obesity, impaired glucose tolerance, dyslipidemia, and hypertension the main features of this condition (11). However recent reports have stressed that NAFLD might be considered an additional component defining MetS both in adults as well as in youth.

This Review aims to provide a general overview of epidemiology, risk factors and pathophysiology of this disease, focusing on current knowledge on the relationship between Obesity, Insulin Resistance, MetS and NAFLD in children and adolescents. In addition, we have summarized the most recent available therapeutic strategies for this disease in young subjects.

## Research Strategy

We proceed with a review of data presented in Literature on NAFLD, with a major focus in obese children and adolescent, through one of the main providers of academic search engines (PubMed). We analyzed articles and Guidelines of major international scientific societies of the last 20 years using as keywords “Non-alcoholic fatty liver disease”, “Fatty liver”, “Liver steatosis”, “Steatohepatitis”, “Obesity in Children”, “Metabolic syndrome”, and “Insulin Resistance”.

## Epidemiology and Risk Factors

Recent data report a prevalence of NAFLD of 3%–10% in children worldwide, with a wide variability depending on exposure to different risk factors (12). In **Table 1** are summarized the main risk factors for NAFLD.

**Table 1:** Risk factors for NAFLD in children and adolescents.

Established risk factors	Suspected risk factors
Physical inactivity	Hypothyroidism
Dietary factors (high cholesterol and saturated fats, high fructose intake, low carbohydrates, no breastfeeding)	Hypopituitarism
Male sex	Hypogonadism
Age (mean age 10–13 years)	Obstructive sleep apnea
Puberty and estrogens deficiency	Polycystic ovarian syndrome
Ethnicity (Hispanics>Caucasian>African-American)	Total parental nutrition
Overweight - Obesity	Rapid weight loss
Insulin Resistance, Abnormal glucose tolerance (prediabetes or diabetes)	Pancreato-duodenal resection
Hypertension	Dysbiosis
Central obesity (waste circumference, visceral fat)	
Lipid metabolism alterations (low HDL-c, high LDL-c and/or total cholesterol)	
Metabolic Syndrome (MetS)	
Genetic factors (PNPLA3, TM6SF2, GCKR, MBOAT, PPP1R3B)	
Microbioma	
Infections (HCV)	
Toxins, drugs and alcohol consumption	

## Obesity and IR

Certainly, one of the factors contributing to the rise of NAFLD diffusion is obesity. In fact, paralleling the epidemic proportion of overweight and obesity in children and adolescents, in the last three decades it has been reported a doubled prevalence of hepatic steatosis with about 7 million children and adolescents affected by NAFLD in USA (13, 14).

According to data provided by the World Health Organization (WHO) in 2016, 18% of children and adolescents aged between 5 and 19 years are overweight or obese worldwide and the forecasts estimate a further increase over the years (15, 16). The epidemiology of NAFLD has strongly suffered from this trend. Epidemiological data on NAFLD established a global prevalence of 7.6% in the general pediatric population and of 34.2 % in obese children (17). Similar data have been reported in a more accurate study published in 2006 that revealed a rate of 38% in autopsy performed in obese children and adolescents (aged 2–19 years) with NAFLD in the USA. The rate decreased in this study to 9.6% prevalence in subjects with normal weight (13). However, despite this positive association, there is not a strict correlation between BMI and NAFLD. In fact, same patients develop NAFLD despite having a BMI value in the range considered as normal (18, 19).

Obesity, hepatic steatosis and IR are three pathological conditions highly correlated to each other likely to be considered part of a general metabolic dysregulation. In fact, it is well-established that children with a definitive diagnosis of NAFLD have a higher prevalence of glucose and lipid metabolism alterations.

To support this statement, different large cohort-studies conducted on adults (20, 21) and adolescents (22–24) have identified IR as an essential factor for liver fat accumulation. Nonetheless, the relationship between IR and hepatic steatosis is still controversial because it is still unknow whether IR is a risk factor or a consequence of fat liver accumulation. The loss of action of insulin in the adipose tissue could be the root cause because of the increased release of fatty acids (FFAs) into blood stream consequent to lipolysis. In fact, it has been demonstrated that 65% triglycerides accumulated in fatty liver originate from circulating FFAs (25). However, D’Adamo et al. (26) have examined the contribution of fat liver content (quantized through Magnetic Resonance Imaging) to induce IR in insulin-sensitive tissue in obese adolescents. They have shown that fatty liver accompanies IR in muscle and liver and the defect in β-cell insulin production. This observation confirm the lipotoxicity theory, according to which fat accumulation in non adipose tissues represents the driven event to IR development (27). Kim et al. (28) conducted a longitudinal study on a multiethnic group of 76 obese adolescents valuing the contribute of baseline HFF (Hepatic fat fraction) to induce IR over the time. They found a persistence of IR in patients with elevated HFF at baseline. This evidence suggests a role of fat hepatic content as determinant of IR.

Intracellular changes could explain the relationship between NAFLD and IR. Autoptic studies conducted in obese adults with signs of IR suggested that not only triglycerides but also other lipid classes might play a role in inducing alterations in transmission in insulin-mediated intracellular signaling (29). Moreover, hepatic inflammation could decrease insulin sensitivity activating proteases which block intracellular pulse transmission (30). However, fat liver accumulation is not necessary linked to IR. In fact, animal studies conducted on mice with alterations in lipid metabolism genes did not show changes in insulin sensitivity (31, 32).

Finally, similarly to adipose tissue and skeletal muscle, studies evaluating animal and human livers have shown alterations in protein expression secondary to fat accumulation, responsible of dysmetabolic state (33–35). In this regard, an upregulation of 69 proteins (hepatokines) in the plasma of subjects with T2DM have been documented. Because of a large part of these effects are also produced during dysregulations of lipidic metabolism, it has been postulated a their possible involvement in the pathophysiological mechanisms linking NAFLD to IR (36).

In the last 20 years, a lot of scientific papers have detected corporeal fat partitioning as the main determinant of IR. Some initial suggestions were provided by Weiss et al. (37) who positively correlated increased intramyocellular lipid content (IMCL) and visceral adiposity (VAS) to impaired glucose tolerance (IGT) in obese adolescents. Caprio et al. (38), examining a multiethnic cohort of obese adolescents, observed a higher rate of IGT and a lower insulin sensitivity in patients with higher VAS rather than subcutaneous fat accumulation (SAT) (39).

There is scientific evidence about the association of insulin-stimulated sterol-regulatory element-binding protein-1c (SREBP1c) pathway in SAT, ectopic fat accumulation and IR. Moreover, Kursawe et al. (40), studying the lipogenic gene expression in 53 adolescents with the same degree of obesity, found that the expression of the carbohydrate-responsive elements binding globulin (ChREBP), a well-defined positive regulator of de novo lipogenesis in adipose tissue, is elevated in the liver and decreased in SAT. These findings could be useful to explain fat liver accumulation in subjects with IR and visceral adiposity.

## NAFLD and MetS

The rising of childhood obesity is accompanied also by an increased prevalence of Metabolic Syndrome (MetS) in children and adolescents. Nowadays, it is estimated that about 40.8% of children with NAFLD presented MetS (41). In contrast to adults, it does not exist a universally accepted definition of MetS in the pediatric population (11, 42, 43). The most recent scientific evidence provided by the International Diabetes Federation (IDF) propose to consider central obesity as the main characteristic of this condition and hypertriglyceridemia, low high-density lipoprotein cholesterol (HDL-C), hypertension, and alteration in glucose metabolism as additional factors. MetS diagnosis can be posed in presence of central adiposity with 2 or more additional factors only in children aged 10-16. For adolescents older than 16 years the IDF suggests using adult criteria for MetS (44), while MetS cannot be diagnosed in those subjects younger than 10 years. These definitions need to be reviewed because some metabolic alterations are already detectable in prepubertal children (45–48). Therefore, the diagnosis of MetS should be suspected in all children and adolescents with risk factors aforementioned.

In Literature, there is a wide evidence about the close connection between NAFLD and MetS in obese population, both in adults (49, 50) and children (11, 51). A case-control study of obese and overweigh children with and without NAFLD showed a significantly higher fasting glucose, insulin, total cholesterol, low-density lipoprotein cholesterol (LDL-c), triglycerides, systolic blood pressure and diastolic blood pressure, and lower HDL-c in children with NAFLD (52). Hampe et al, examining 77 obese non-diabetic children, showed a direct correlation between ALT levels and numbers of criteria for MetS. In addition, by evaluating MRI obtained data on Hepatic Fat Fraction in obese adolescents, it has been shown that children with HFF greater than 5.5 % have a tripled risk to develop MetS (53).

NAFLD is an independent risk factor for T2DM and probably also for cardiovascular disease in adulthood (6, 7, 54–55). About 8%–10% of children with recent diagnosis of NAFLD are diabetic and, on the other hand, 50% of children with T2DM have elevated ALT serum levels indicative of NAFLD (52).

A cross-sectional cohort study published in 2019 have provided data on current distribution of alteration of glucose metabolism in Caucasian children and adolescents with a biopsy-proven diagnosis of NAFLD. Results have shown that 124 out of 599 (20.6%) subjects featured abnormal glucose tolerance of which only 0.8% (5/599) with a diagnosis of T2DM, while 19.8% (119/599) prediabetes (impaired fasting glycemia or IGT). There were no differences between sexes (56). These statistics are different from those derived from a multi-ethnic cohort of US children/adolescents with NAFLD enrolled in the NASH Clinical Research Network. In fact, the prevalence reported by authors was of 6.5% for T2DM and 23.4% for prediabetes (57). Probably, this last study has been influenced by ethnic variability. In fact, a more severe alteration in glucose homeostasis is well known in black obese adolescents than whites and Hispanics, thus partially explaining these differences (58).

The linkage between fatty liver and hypertension has been well presented in a systematic longitudinal study conducted on 382 obese pediatric patients. At the time of diagnosis 36% of them had elevated blood pressure. Authors also described a strict correlation between severity of the disease and the probabilities to have hypertension (59).

Alteration in lipid profile is another feature of children with NAFLD. In Literature, elevated non-HDL-C levels and increased TG/HDL-C ratio are reported as risk factors of IR and NAFLD in children and adolescents (60). In addition, in 2010, Nobili et al. (61) showed a positive correlation between severity of histology and atherogenic profile. All these metabolic alterations translate in an increased cardiovascular risk in subjects with NAFLD. Therefore, the comprehension of the relationship between NAFLD and MetS could be useful in order to offer a prognostic point of view in defining the risk of cardiovascular diseases in these group of children and adolescents. Studies evaluating adult subjects reported a close association of Mets and NASH and hepatic fibrosis (50, 62). Recently, in an obese pediatric sample with NAFLD, Patton et al. have confirmed the presence of more severe histological phenotype in patients with almost three criteria of MetS and have proposed central obesity and IR as factors most consistently associated with NAFLD histology (63).

Pathogenetic mechanisms related to the association of NAFLD and MetS are not yet known. Certainly, IR have a pivotal role in inducing metabolic alterations in common between NAFLD and MetS, but it has emerged a possible contribute of additional factors and particularly of genetic profile and environmental factors (64, 65).

## The Role of Age, Gender, and Ethnicity

Currently, the individuals suffering the most of NAFLD in youths are those aged 10–13 years (66). In fact, several studies report a mean age of 12 to 13 years at diagnosis (59, 63, 67), but Italian case reports suggest a lower age of onset (10 to 11 years) (22, 68).

Similar to adult subjects, in the pediatric population there is a direct correlation between NAFLD prevalence and age (69–71). In 2008, the Study of Child and Adolescent Liver Epidemiology (SCALE), conducted on children aged 2–19 years, showed a growing rate from 0.7% in children 2–4 years old to 7% in teenagers (13). This evidence is in accord with the rising trend of overweight and obese children with advancing age (72). The use of ALT is enabled to identify elevated levels of this marker also in preschool-aged children with a percentage of 26% of obese children aged 2 to 5 years in Chicago (73) and 15% of Hispanic children 4 to 5-year-old in Houston (74). Although, it is still unknown the proper meaning of these results, those preliminary data surely represent a red flag for a greater involvement of pediatric population in the future.

Puberty represents a key moment for the onset and progression of obesity due to Insulin Resistance development. Amiel et al. (75) described a lower insulin sensitivity in pubertal young than pre-pubertal children and adults. Later, this evidence has been validated by cross-sectional and longitudinal studies (76–78). The most important confirmation has been provided by Moran et al. (79). They revealed a reduction in insulin sensitivity (valued through hyperinsulinemic euglycemic clam) starting from subjects with Tanner 2 with nadir in mid-puberty (T3/T4) and recovery when the Tanner stage 5 is achieved. However, a study conducted in 2007 has observed a prevalence of hepatic steatosis of 52% in obese prepuberal children. Furthermore, it has been pointed out a higher involvement in hepatic steatosis in older children, presuming a possible role of duration of obesity (46). Hepatic and peripheral IR seem also to increase during puberty (63), thus combining their effect during this age. It is not completely clear the relationship between obesity and IR during puberty. Certainly the decline of insulin sensitivity during puberty is independent from obesity (80), but in obese youth IR persists over time with negative impact on metabolic balance (81, 82). However, according to previous evidences (23, 83), two different studies have shown that metabolic abnormalities related to IR are already observed either in children aged 6–10 years without clinical signs of pubertal development (15) or younger (45).

Data from published case reports suggest a doubled risk to develop NAFLD for male sex (20, 21). Not surprisingly male subjects tend to have a higher visceral abdominal adiposity, which correlates positively with IR and NAFLD. A key role could be played by metabolically active androgens. In fact, individuals with IR have decreased production of sex hormone-binding globulin and women with polycystic ovary syndrome (sustained by IR) have elevated levels of free androgens with a subsequent higher risk to develop NAFLD than the general population (84). Besides, in ovariectomized female mice, the reduced production of estradiol promotes fat liver accumulation (85). Finally, the role of sex hormones is also underlined by the absence of gender difference during prepuberty (46).

There is a wide diversity of prevalence among different regions around the world. In the Western nations, paralleling the high rate of obesity and T2DM, almost one-third of the population is involved in this disease (86). However, the actual westernization process in developing countries is leading to an alarming increase of obesity and its correlated comorbidities, especially NAFLD (15).

South American and Asian countries are affected the most in the world with a reported prevalence of 25%, while Africa is affected by less than 5 % (12, 87). Simultaneously the economic growth, the rate obesity and metabolic syndrome has increased in China with a subsequent achievement of NAFLD prevalence of 68.2% in obese children, over the last two decades (88). The rate of prevalence in India varied from 16% to 32%, with a significant reduction in the rural part of the state affecting only the 9% of subjects (89). Nevertheless, NAFLD prevalence varies within a region, despite the exposition to the same risk factors. It is believed that ethnicity plays an important role in the epidemiologic variability both in children (13, 90) and adults (91, 92), by several mechanisms. Firstly, race might influence the severity of obesity. Shinner et al., analyzing epidemiological data of the USA population from 1999 to 2012, have reported a prevalence of severe obesity of 10% in non-Hispanic whites, 20% in non-Hispanic blacks, and 16% in Mexican Americans. Also, it has been shown how Hispanic girls and non-Hispanic black boys tend to have higher levels of adiposity severity (93). A study published in 2004 based on a multiethnic population sample of 2,287 subjects (32.1% white, 48.3% black, and 17.5% Hispanic) showed a higher prevalence of NAFLD in Hispanic (45%), followed by Caucasian (33%), and African-American (24%) (91). The estimated risk of hepatic steatosis among Hispanic adolescents is 4-fold higher than other races (94). It has been reported that black youths do not accumulate triglycerides as the same amount that Hispanics and whites, but when NAFLD developing a more severe metabolic phenotype is observed (58). The explanation of this phenomenon is not related to IR but to different body fat distribution. African-American people react to IR with a higher fat accumulation in subcutaneous tissue rather than visceral adiposity, the latter directly correlated to triglycerides fatty content (95). These racial differences are useful to understand how interactions between different factors (genetics, physiology, culture, socioeconomic status, and environments) can promote the onset and the progression of the disease.

## Genetic Background

The extreme variability in the prevalence of NAFLD existing between different groups of the same region exposed to the similar risk factors suggests a possible role of a genetic predisposition. To support this hypothesis there is evidence of a certain degree of hereditability. In fact, 59% of siblings and 78% of parents of obese children with biopsy-proven NAFLD develop hepatic steatosis with a rate of hereditability of 1.0 for fatty liver and 0.386 for liver fat fraction (3). The risk to develop the progressive form of NAFLD increases to 12 times in family members of patients affected by NAFLD-related cirrhosis (96). Moreover, in a study evaluating a population of twins, Loomba et al. showed a correlation between hepatic steatosis and liver fibrosis in monozygotic twins, but not between dizygotic twins, thus presuming a hereditable trait (97). A genetic background could also explain the variability in fat liver content existing between different races. In fact, Hispanic Americans develop NAFLD with higher frequency than African Americans, despite the same degree of IR and obesity.

The technological advancement in genetic has allowed to identify a lot of genes correlated to the risk of developing and progression of MetS and NAFLD both in children and adults (98–100). The focus was mainly on genes involved in lipid metabolism, oxidative stress, insulin signaling, and fibrogenesis. In a recent study in 514 caucasian obese children and adolescents, authors examined the contribute of genetic variants in determining changes in clinical risk. Particularly, eleven single nucleotide polymorphisms (SNPs) have been examined and authors have proved that three of them (PNPLA3 rs738409, TM6SF2 rs58542926, and GCKR rs1260326) are strongly associated to hepatic steatosis in children; while the variant ELOVL2 rs2236212 have a modest connection with NAFLD development, independently of age, sex, and z-BMI (101). These same polymorphic variants have been studied by Morandi et al. with the aim to establish their role in IR in obese children and adolescents and nondiabetic adults (102). Despite authors have not observed any significant evidence that genetically-influenced NAFLD is causally associated with IR, this study have suggested some explanation of many unknown pathogenetic aspects which need to be further explored.

The single-nucleotide polymorphism (SNP) common variant allele (rs738409) in patatin-like phospholipase domain–containing protein 3 (PNPLA3) has been previously described conferring susceptibility to NAFLD both in adults (103) and in children (68). As the result of changes of isoleucine to methionine at codon 148 (I148M), studies *in vitro* have showed that the adiponutrin synthesized surrenders its usual lipolytic function and increases its lipogenic activity with subsequent triglycerides accumulation both in the adipose tissue and in the liver (104). Perttilä et al. (105) investigated transcriptional regulation of PNPLA3 induced by glucose in an immortalized human hepatocyte (IHH) cell line. They found that ChREBP, a key regulator of glucose metabolism and fat storage, is the mediator of transcriptional induction of PNPLA3 gene. In addition, authors showed that FFAs improved further the effect of variant I148M, thus promoting TG accumulation. According to the last scientific evidences, obese children with MetS, carriers of this variant, have elevated ALT levels than general pediatric population (106, 107) and the risk to develop NAFLD is 1.3- to 2.4-fold increased if compared with weight-matched subjects homozygous for normal allelic variant (108, 109).

The single nucleotide polymorphism rs58542926 C>T in transmembrane 6 superfamily member 2 (TM6SF2) is another genetic variant associated to an increased risk to develop hepatic steatosis and fibrosis in children (110). The Glu167Lys (E167K) variant encodes for a protein that decreases VLDL-mediated lipid secretion favoring hepatic fat accumulation (111). In 2014, Zhou et al. (112) assessed how the E167K variant in TM6SF2 influenced metabolic profile. They showed that patients with this polymorphism are characterized by a distinct phenotype dominated by a preserved insulin sensitivity of lipolysis and hepatic glucose production. Furthermore, they described lower circulating TG levels despite an increased liver fat content. It could be translated in a reduction in cardiovascular risk (113).

The s1260326 Glucokinase regulator (GCKR) variant has been described associated to NAFLD and alteration in lipid profile such as elevated triglycerides and large VLDL levels in obese children (114). The mutated protein characterized by proline-to-leucine substitution does not respond to inhibitory stimulus of fructose 6 phosphate, thus resulting in a continuous activation of gluconeogenesis. An increased GCK activity may lead to increased glycolytic flux, increasing Malonyl CoA, precursor of TG through intra-hepatic De Novo Lipogenesis (115).

Notably, it has been described a lower frequency of the SNP rs738409 in the PNPLA3 and rs1260326 in the GCKR in blacks than Hispanics (90, 116). Considering that Hispanic ethnicity was also associated with younger age at biopsy it could be postulated an anticipation of phenotype in subjects carrying the genetic risk (117). This evidence could justify a screening program at a younger age for these subjects.

ELOVL2 is a newly discovered genetic variant identified through genome-wide association studies. The gene product is an elongase which catalyzes the reaction of synthesis of polyunsaturated fatty acids (n-3 PUFAs). Particularly, an inverse correlation between this gene and plasma n-3 PUFA level has been observed (118), thus it may partially contribute to genetic risk of NAFLD development (101).

Moreover, other genes described as associated to an increased risk of NAFLD are the mutation of membrane bound O-acyl transferase 7 (MBOAT) and the protein phosphatase 1 regulatory subunit 3B (PPP1R3B). The MBOAT7 could alter the remodeling of phospholipids, thus inducing their accumulation (119). One single genetic variant of the gene encoding the PPP1R3B has been described as protective against NAFLD. In fact, this variant promotes glycogen synthesis instead of lipogenesis.

However, genetics alone cannot explain the extreme variability in the prevalence of the disease. Schwimmer et al. attempted to characterize the heritability estimates with 0 being no heritability and 1 representing a trait that is completely heritable. They showed a genetic effect roughly of 0.85 considering NAFLD as unadjusted dichotomous variable. In contrast, this estimation decreases to 0.58 if hepatic fat fraction (HFF) is used as a continuous variable (52). Probably, the onset and progression of NAFLD are mediated by the interaction of multiple factors, as recent works have also demonstrated (120). Given the variety and number of genes involved in NAFLD, scientific society is putting forward the idea of using genetic risk scores to predict NAFLD development and progression in which clinical risk factors are combined with genetic profile. A first attempt has been made by Nobili et al. (121) who demonstrated the superiority of a combined genetical 4-polymorphism risk score (including rs738409, rs4880, rs3750861, and rs13412852 variants) and clinical score compared to a clinical risk score alone (including age, diastolic blood pressure and serum AST levels) in predicting progression towards end-stage liver diseases. Recently, Zusi and colleagues (101) have shown an increased capacity to predict NAFLD development of 5.2% for a genetical risk score characterized by the association of clinical and genetical risk factors than clinical risk score. These models imply on a side the possible additional role of different allelic genetic variants predicting NAFLD development. In addition, on the other side they create the basis to establish an individual risk of disease.

In conclusion, a more careful analysis should be programmed to understand the relationship between environmental factors and genetic background.

## Lifestyle and Diet Quality Influence

**Figure 1:**
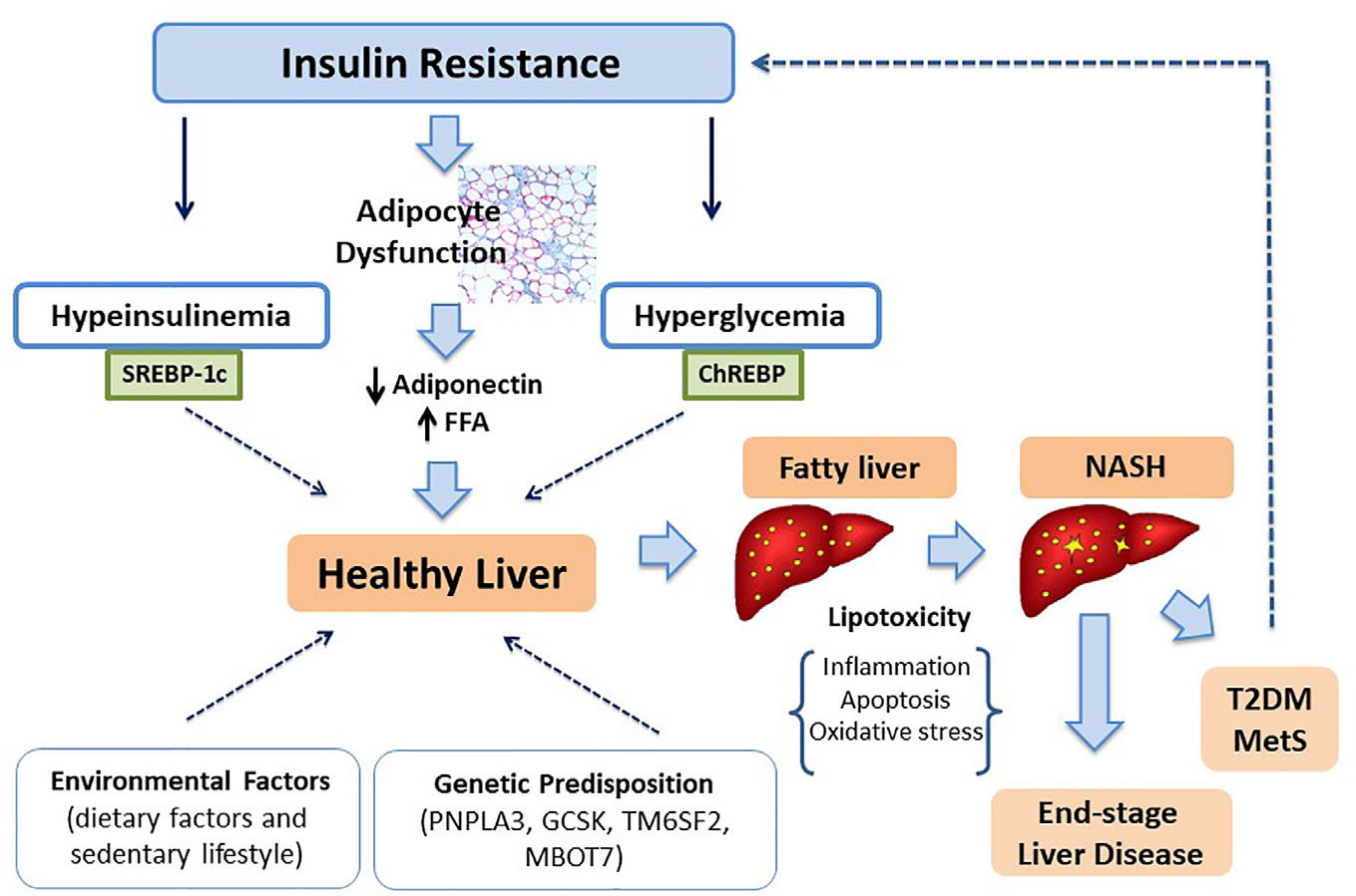
The role of IR in NAFLD development and progression.

Age, sex, and ethnicity combined with genetic predisposition represent the main risk components which promote fat accumulation. However, environmental and individual factors could affect the natural history of liver damage and metabolic dysregulation (122, 123) (**Figure 1**). Certainly, high-fat diet and sedentary lifestyle, contributing to weight gain, result in increased body fat deposition and thus to an increased risk to develop NAFLD as well as to end-stage liver diseases (124, 125). Population studies in adult patients have identify a diet mainly based on carbohydrates, saturated fat (SFA) and cholesterol rich foods in patients with NAFLD (126). Feldestein et al. (127) have suggested that SFAs increase apoptotic cell death, liver injury and promote progression toward NASH independently from TG hepatic content. In contrast, unsaturated FFAs conduct to elevated TG accumulation, but they not significantly affect cell vitality (128). It has been observed that an elevated omega-6/omega-3polyunsaturated fatty acids (PUFA) ratio in dietary intake is a risk factor to metabolic alterations and NAFLD (129, 130). Santoro et al. identified oxidized linoleic acid (LA) metabolites (OXLAMs) as possible mediators of hepatic inflammation (131). In fact, they regulate peroxisome proliferator-activated receptor (PPAR)-alpha with subsequent increased lipid transport and oxidation, predisposing to hepatic inflammation and NASH. Add to this, they reported a direct correlation between oxidized lipids (OXFAs) levels and the spectrum of glucose tolerance. Great importance it is given to fructose consumption in children. Mosca et al. evidenced an impaired metabolic profile in children receiving elevated amount of fructose, thus presuming its possible role in the pathogenesis of hepatic steatosis (132). Furthermore, Nobili et al. showed a direct correlation between dietary fructose intake and evolution towards NASH in a sample of children and adolescents with a histologically confirmed diagnosis of NAFLD (133). At the same time, the restriction of fructose intake is associated to a reduction in liver fat content and lipogenesis (134).

Some authors have observed that the exposure to environmental factors in utero could increase the risk to develop NAFLD later in life (135). In fact several studies have clearly shown that both low and high birth weight are associated to elevated possibility of severe hepatic steatosis (136, 137). Moreover, it has been shown a higher mean HFF, as defined by magnetic resonance spectroscopy) and hepatic steatosis in infant born to mothers with gestational diabetes and diabetes mellitus, respectively (138, 139). In contrast, after birth, the exclusive breastfeeding is preventive to NAFLD development and progression to NASH (140, 141). Furthermore, an autoptic study conducted on 105 mothers matched to their neonates have shown a positive correlation between BMI value at conception and HFF in neonates (142).

Recent advances have attributed to epigenetic changes a in the complex relationship between environment and genetic predisposition, also in prenatal age (143). The term epigenetic indicates any modifications of DNA capable to alter genic expression including DNA methylation, modifications in histone proteins and by micro RNAs (miR) (144). It has been described a strong relationship between nutrition and DNA methylation. In fact, a diet poor of methyl donors (folate, choline, and betaine) could impair the normal methylation of genes normally silenced. Therefore, these effects result in an abnormal expression of genes involved into DNA damage and repair, lipid and glucose metabolism, fibrosis, and liver remodeling (145). Another epigenetic mechanism involved into increased risk of NAFLD development is the unbalanced acetylation/deacetylation of histone proteins (146). In fact, SIRT1, a sirtuin member of histone deacetylaces, have been reported to be involved in metabolic homeostasis regulating fatty acid oxidation, gluconeogenesis, lipolysis, and mitochondrial activity (147). Moreover, this protein inhibits NF-κB action thus modulating inflammatory response. Therefore, its absence in liver could be associated to NAFLD damage progression (148). It has been shown that an overexpression of histone acetyltransferase (HAT) activator p300, linked to NAFLD progression, determines hyperacetylation of carbohydrate-responsive element-binding protein (ChREBP), promoting its transcriptional activity. In addition, this epigenetic alteration has been described related to liver lipogenesis in mice and was associated with IR (149). There is much still to learn about mi-RNA involvement in increasing NAFLD risk and progression. They regulate genetic expression linking to complementary sequences on mRNA. Although some mi-RNA has been described as elevated in patients with NAFLD (150, 151), their role in hepatis steatosis needs to be clarified (152).

In conclusion, epigenetic changes increase the interindividual risk variability making more complicated the possibility to identify an individual risk profile.

## Microbiota

Over the last years, the attention of the scientific community has been focused on a possible role of gut microbiota on the pathogenesis of many diseases, and particularly NAFLD.

The postulated hypothesis is that changes in gut microbiota composition or alterations in its functionality contribute to metabolic imbalances in adipose tissue, muscle, and liver (153–155). Bacteria composition of intestinal microbiota is influenced by different factors, including both local factors (intestinal acidity, mucus) and extrinsic factors such as drugs and diet (156–158). In relationship to dietary intake, bacteria localized in proximal trait of intestine use mainly non-digestible carbohydrates as source of nourishment through saccharolytic fermentation with production of short chain fatty acids (SCFAs) acetate, propionate and butyrate (159, 160). Conversely, the progressive consumption of carbohydrates is responsible of a switch towards a proteolytic fermentation. The degradation of branched-chain amino acids (BCAAs) results in production of gaseous products such as hydrogen, methane, carbon dioxide and hydrogen sulfide but also branched chain fatty acids (BCFAs) isobutyrate, 2-methylbutyrate and isovalerate. Instead, the fermentation of aromatic amino acids (AAAs) is involved into the production of phenolic and indolic compounds (161).

From an analysis of Literature, it has emerged that the proteolytic fermentation is associated to an increased risk of NAFLD than saccharolytic fermentation (162). In fact, studies in rodents have shown a beneficial effect of butyrate to regulate the gut-liver axis by increasing tight junction stability and limiting the afflux of pro-inflammatory mediators to the liver in rodens fed with an high fat diet (163, 164). Moreover, studies *in vivo* on animal model have bring to light a reduction in hepatic fat accumulation after administration of acetate, propionate or butyrate (165–170). In contrast, some metabolites derived from proteolitic fermentation have been associated to a proinflammatory state caused by the alteration in epithelial permeability responsible of exposition of hepatic parenchyma to toxic substances (171, 172). A very interesting study conducted on mice free from intestinal germs has confirmed the implication of gut microbiota in NAFLD. It demonstrated that intestinal transplantation of germs derived from diabetic mice combined with a high fat diet leads to macrovesicular hepatic steatosis. By contrast, a low-grade hepatic steatosis is observed after transplantation of intestinal microbes derived from healthy mice (173).

On the basis of these findings, an alimentation based mainly on dietary fiber and poor in proteolytic products should be considered as a possible therapeutical approach to prevent and/or treat children affected with metabolic alterations (174).

In term of microbial population, human studies have not found an association with a particular pattern of intestinal germs. Some authors have proposed a role of Bacterioides and Firmicutes in the NAFLD progression, but available data are currently inconsistent (175, 176). Greatest information might be obtained evaluating the protective/therapeutical effect of prebiotics and prebiotics. In fact, oral supplementation of fructo-oligosaccharide for 8 weeks has been associated to ALT, AST, and insulin reduction in patients with NAFLD (177). Moreover, the simultaneous administration of fructo-oligosaccharide and *Bifidobacterium longum* was associated with a reduction in steatosis, NASH activity index, serum levels of TNF and of endotoxin after 24 weeks (178).

In conclusion, different clinical studies have attempted to find in microbioma the key to characterize subjects at increased risk to develop NAFLD and to progress to end-stage liver disease. The aim is to identify biomarkers for diagnosis and prognosis of disease, but further evaluations and a major progression in diagnostic methods are necessary to achieve this goal (179).

## Pathogenesis and End-Stage Liver Diseases

The term NAFLD includes a large group of clinical and histological conditions ranging from simple triglycerides accumulation in more than 5% of hepatocytes to non-alcoholic steatohepatitis (NASH), characterized by steatosis, hepatocellular ballooning, and variable degree of lobular inflammation and fibrosis.

Currently, despite considerable progress in identifying possible mediators of intrahepatic fat accumulation, the processes underling liver damage are still poorly understood.

In 1998 Day and James (180) postulated the “two-hit hypothesis” of NASH originated by the observation that steatohepatitis could be inducted with injections of bacterial lipopolysaccharide in hepatic cell of mouse with steatosis caused by high-fat diet or by mutation in leptin receptor (mouse ob/ob) (181, 182). According to this theory, the “first hit” is represented by fat accumulation leading to steatosis, while the “second hit” includes pro-inflammatory stimuli (oxidative stress, inflammatory cytokines, adipokines, mitochondrial dysfunction, endoplasmic reticulum stress, and gut-derived bacterial endotossine) which determine necro-inflammation and fibrosis leading to end-stage of disease (183, 184). However, based on recent knowledge on the interaction of environmental factors and genetic, it has ended up to novel models and particularly to the “multiple hit model” whereas by the interactions of multiple factors bring to different clinical and histological phenotype (185). In this model, IR plays a pivotal role. Particularly, the loss of insulin regulatory action of lipolysis in adipose tissue and of hepatic glucose production leading to an increased flux of free fatty acids toward liver. These alterations, in association with higher levels of intrahepatic glucose, promote lipogenesis thus resulting in accumulation of TG inside hepatocytes. TG are not harmful for hepatic cells, but they protect hepatocytes against FFA-induced damage (186, 187). In fact, elevated FFA levels and their accumulation in non-adipose cells can induce cell dysfunction (lipotoxicity) and apoptotic cell death (lipoapotosis). The mechanisms involved seem to be the increased mitochondrial and peroxisomal β-oxidation, producing reactive oxygen species. The local proinflammatory state is responsible not only of hepatic injury but also of overproduction of proinflammatory cytokines. The alteration of cytokine signaling-3 action leads to persistent hyperinsulinemia, which further worsens IR stimulating the SREBP1c that enhances lipogenic genes expression and reduces fatty oxidation (188).

Although with a lower frequency, also children can progress towards steatohepatitis to end-stage liver diseases such as cirrhosis and hepatocellular carcinoma. Schwimmer et al. (13) reported a NASH prevalence of 2.6% in the general pediatric population. However, the prevalence rises to 23% in children with NAFLD. In addition, fibrosis and/or cirrhosis were present in 9% of children with NASH. By 2030, the prevalence of end-stage liver disease is estimated to rise by 50% in Western nations and Asian countries (86).

It is necessary to investigate on several factors that promote the progression towards the various stages. Certainly, NASH is predominant in children and adolescents with prediabetes or diabetes (57) and the contemporary presence of biopsy-proven NAFLD and altered glucose tolerance has been described as factor that increases 2-time the risk non-alcoholic steatohepatitis (56). Once again, ethnicity plays an importance role because it has been reported that African-American children develop NASH with less tendency (189).

The natural history of the disease in pediatric age is not understood because there are not adequate number of longitudinal studies about this condition. A 2-year randomized control trial (TONIC trial) evaluated changes in histology at baseline and at 2-year follow-up in three groups of patients treated with vitamin E, metformin, and placebo, with the same advices for the three groups. In the placebo arm, 28% showed a resolution of NASH, 40% improved fibrosis, 40% improved steatosis, and 43% improved lobular inflammation. Advanced fibrosis characterized 17% of obese children with NAFLD referred to liver center after screening (190). Considering the long-life expectancy, these subjects are more predisposing to develop long-term complication such as cirrhosis, liver failure, and hepatocellular carcinoma than general population (191, 192).

In adult patients, NAFLD is associated an increased risk to develop hepatocellular carcinoma (HCC). In children this possibility is rare, although reported also in this age (193) and it is expected a further increase paralleling the increased number of young people with NAFLD/NASH. However, all patients with NAFLD should be followed to evaluate the risk to develop HCC in adult age. A longitudinal study conducted on a cohort of 280,000 children aged 7 to 13 years affected by NAFLD, identified a correlation between BMI z-score and the risk to develop HCC. Particularly, results of this study have shown that for each BMI z-score increase of +1, the risk of HCC increases of 33% (190).

It has been described a more rapid evolution of NAFLD in children than in adults with an increased mortality and morbidity (194). These considerations have led during the last year to consider that the pediatric NAFLD could represent an aggressive type of NAFLD. This statement needs to be validated with prolonged longitudinal studied evaluating the effects of an early exposition to the risk factors of NAFLD.

The comprehension of the pathogenetic mechanisms leading to different degree of histological damages should be implemented with the aim to identify the key moments of this complex mechanisms. Certainly, the emerging of genetic susceptibility will improve our understanding of the disease and will allow us to guide treatment with a subsequent prognosis improvement.

## Diagnosis

Unfortunately, there is not a specific test for NAFLD. Therefore, it remains a diagnosis of exclusion (1). For an adequate diagnostic evaluation, an initial assessment of personal and family medical history and physical exam are necessary. Given the strong relationship between NAFLD and visceral adiposity, the measurement of waist circumference and BMI should be the first step in the physical examination, together with assessment of pubertal development.

In clinical practice, ALT levels are widely used as a surrogate marker of NAFLD in the pediatric population because of its good correlation to the prevalence of NAFLD (192). In fact, after excluding secondary causes of ALT elevation, increased levels are predictive of NAFLD in children. By using ALT as a diagnostic test, the National Health and Nutrition Examination Surveys (NHANES) in the USA has reported a NAFLD prevalence of 6%–11.5% among adolescents, tripled from 3.9% in 1988–1994 to 10.7% in 2007–2010 (195). Similar data have been described in cohorts from Europe, Korea, Japan, and China, with a significantly higher prevalence also in other countries such as in India (196–200).

However, one of the main limits in using ALT as a laboratory screening test for NAFLD is the lack of a universally accepted threshold because of its variability related to age, gender, ethnicity, and lifestyle (201). The European and North American Society for Pediatric Gastroenterology Hepatology and Nutrition suggest using sex-specific upper limits of normal in children (22 U/L for girls and 26 U/L for boys) (1, 2). NASH is more common in children with ALT upper to 80 U/L compared to those with ALT <80 U/L (41% compared to 21%, respectively) (191). However, ALT levels may not be related to the degree of histological damage.

Ultrasonography (US) is another diagnostic tool widely used as screening exam in pediatric patients mainly due to some of its related advantages and particularly its non-invasiveness, widely diffusion and cheapness (202). The prevalence of NAFLD in healthy European adolescents evaluated with ultrasonography was assessed to be 1.8% (203). The rate of prevalence increases to 60% in children and young people undergoing bariatric surgery until to 80% in those obese (87, 204). This technique allows physicians to identify fatty infiltration through the echogenicity degree (9). Physiologically, hepatic parenchyma has the same echogenicity of the surrounding organs, in particular the kidney. The presence of steatosis leads to liver “brightness” or hyperechogenicity. When liver fat infiltration involves more than 20% of hepatocytes, US is capable to detect steatosis with a sensitivity and specificity of 100% and 90%, respectively (205, 206). Furthermore, it is a useful tool to identify signs of portal hypertension (207). However, US does not support the diagnosis or grading of hepatic steatosis in children and, although a paper published in 1997 gave a greater diagnostic power to the US (208), recent evidence suggests a comparable diagnostic accuracy between ALT and US, with a moderate capacity to detect pathological fat accumulation (209). Therefore, it is currently used as screening test (210) The inability to distinguish between a simple steatosis from steatohepatitis and to quantify fatty infiltration are the main limits correlated to this imaging method (211).

Currently, Expert Committee guidelines recommend using both ALT and US for the screening of NAFLD in children and adults (212).

Clinical, biochemical, and imaging evaluations are of value in the initial assessment of patients with NAFLD, but liver biopsy remains the most sensitive and specific test to establish the definitive diagnosis and to stage and grade the disease (49). It is the only diagnostic tool capable to detect histological features of NAFLD (steatosis, hepatocyte ballooning, inflammation, and fibrosis), but its limits are related to the invasiveness of the procedure which can result in pain, internal bleeding, leak of bile from the liver or gallbladder, and pneumothorax (213). In addition, liver biopsy is also subject to sampling errors related to the histological sample site that does not include damaged areas, beyond the inter-observer and intra-observer variability in the histological pathologist interpretation (214, 215). Considering the low prevalence of NASH in pediatric population, there are specific indications for liver biopsy in obese children and adolescents (**Table 2**) (1). Clinical signs suggestive of increased risk to develop fibrosis in children with NASH may include higher ALT (>80 U/L), splenomegaly, and AST/ALT >1, while panhypopituitarism and T2DM are accepted clinical risk factors for NASH and advanced fibrosis (2).

**Table 2:** Indications for Liver Biopsy of the ESPGHAN Hepatology Committee.

Approach to Liver Biopsy in Children and Adolescents
1. Assessment of NAFLD in children who have increased risk of NASH and/or advanced fibrosis
2. The exclusion of other treatable disease, only if other non-invasive investigations have not been conclusive
3. Before pharmacological/surgical treatment
4. As part of a structured intervention protocol or clinical research trial

In clinical research, two imaging techniques and particularly the 1H-nuclear magnetic resonance [1H-NMR] and the fast magnetic resonance imaging [fast MRI] have offered good perspective with the aim to detect and quantify fatty liver content (23, 37, 216). Liver steatosis is characterized by a hepatic fat content (HFF) up to 5%. However, these techniques do not allow to grade and stage (28).

A lot of techniques are available for the evaluation of liver fibrosis degree. The transient elastography (FibroScan) has been described as a good diagnostic procedure both in adults (217) and children (218) with advanced NAFLD, using liver stiffness as parameter of fibrosis. Despite the availability of a probe with size appropriate for children, it is not widely performed in pediatric clinical practice (219).

The magnetic resonance elastography (MRE) uses a modified phase-contrast MRI sequence to visualize propagating share waves in tissues. It could be a useful technique if used with MRS to evaluate the degree of steatosis and of liver stiffness. However, further investigations should be made in medical research before they can be used in clinical practice.

Several studies have been conducted in adults to identify markers (103, 220) related to propensity to accumulate fat in the liver and subsequently leading towards more deleterious stages of NAFLD/NASH and its associated metabolic and cardiovascular complications. Their identification is necessary to detect at an early-stage hepatic involvement with a non-invasive method. FGF-21 has been selected as possible circulating marker of fatty liver in adults. In particular, Giannini et al. have attempted to characterize this correlation by conducting a case-control study where FGF-21 levels have been in obese adolescents compared to healthy controls (221). The results showed higher plasma FGF-21 levels in obese youth, especially in those with fatty liver, independently from BMI, visceral fat, and insulin sensitivity.

Caspase-cleaved CK-18 is one of the most studied markers for NASH. It is a product of apoptosis releases into the bloodstream after cell death. Considering the strong relationship between apoptosis and NASH, it has been proposed as diagnostic tool. Studies in adult (222, 223) have described a specificity and sensitivity respectively of 94% and 90.5% using as cutoff value 380 U/L. In addition, Kim et al. (28) have shown a positive correlation between ALT levels, percentage of HFF and CK-18 levels in obese adolescents.

Before we can use this biomarker, further cross-sectional and longitudinal studies need to be done to obtain more data about its reliability.

Recently, Khusial et al. (224) tried to identify a screening panel of both metabolomics and clinical features for screening of NAFLD in youth. However, further analysis and validation testing in other cohorts are necessary.

## Treatment

The most important goal of treatment is the regression of NAFLD, defined as a decrease in steatosis, inflammation, and/or fibrosis, while the secondary aim is the resolution of NASH (2). However, we should also remember that NAFLD is part of metabolic syndrome, so visceral fat reduction and IR improvement need to be considered as key goals of NAFLD treatment in children and adolescents.

Currently, there are not a standard and universally accepted therapeutic options for the treatment of patients with NAFLD. Progressive weight loss and regular physical exercise should be the first indications for obese children with NAFLD. In fact, it has been evidenced a positive correlation between western dietary pattern (valued through a questionnaire) and the risk to present NAFLD. Non casually, it has been showed in adult patients that the loss of almost 3-5% of body weight is associated to a reduction in hepatic steatosis, while a higher weigh loss (up to 10%) is associated to histological improvement in the course of steatohepatitis (225).

Although there are not specific recommendations about the type of diet some studies have shown some results. Ramon-Krauel et al. (226) compared the efficacy of a low-glycemic diet or a low- fat diet in two small groups of obese patients (17 cases for group) with NAFLD. They showed the absence of significant differences and comparable decreases of both ALT levels and HFF (valued through MRI) in the two groups after 6 months of diet. The American Heart Association (AHA) suggests a balanced diet with adequate amounts of fruits, vegetables, low saturated fats, fish, with a low fructose and salt intake, with almost 60 min of moderate daily physical activity in patients with NAFLD (227, 228). Drinks and foods containing high fructose concentrations should be limited (2) as well as trans-fats, with an adequate balance between omega-6 to omega-3 polyunsaturated fatty acids (229). It would be desirable to promote a family therapy including cognitive behavioral therapy, outpatient after care with the physician, ongoing face-to-face family therapy and problem-solving approaches (230). The goal is to bring a change in patient’s lifestyle and to obtain weight loss and subsequent reduction in visceral adiposity. The measurement of waist circumference over the time could be a useful parameter to evaluate the benefit of this intervention strategy (231).

However, adolescents usually manifest a refusal of their own illness and a scarce compliance to follow the advices given by doctors. For this reason, approaches adopting diet and physical exercise have often shown only to fail in this group of patients (232).

Currently, there are not available medications proven to treat pediatric patients with NAFLD. During the last years, the scientific research has been focusing on the effect of pharmacological treatments as intervention strategy in adult and pediatric population. Lavine et al. (190) evaluated the benefits of Vitamin E and metformin in a multicenter randomized placebo- controlled trial conducted on children and adolescents aged 8–17 years. The results obtained did not show a substantial reduction in ALT levels both with the use of Vitamin E and metformin. Nevertheless, antioxidant effects of Vitamin E should play a pivotal key to slow NASH progression towards end-stage diseases despite placebo group. Conversely, patients treated with metformin featured a little improvement in histology damage than placebo group. D’Adamo et al. (233) studied the effect of Vitamin E supplementation in 42 obese Caucasian pre-pubertal children (16 boys and 23 girls) affected by liver steatosis. Authors have shown decreased oxidative stress markers [decreased PGF-2 α levels, increased levels of endogenous secretory receptor for advanced glycation end products (esRAGE), improved insulin sensitivity and lipid profile] in obese subjects treated with Vitamin E supplementation (600 mg/day) and lifestyle intervention for 6 months compared to patients managed only with lifestyle intervention. Therefore, Vitamin E supplementation implemented at an early stage of the disease may help in reducing the metabolic and cardiovascular alterations associated with NAFLD in this age group. The American Association for the Study of Liver Diseases (AASLD) guidelines support the use of vitamin E but not metformin in obese children with biopsy-proven NASH (234).

Several studies (235–238) evaluated the possible beneficial effect of using omega-3 fatty acids docosahexaenoic acid (DHA) and eicosapentaenoic acid in improving lipid metabolism in children. In fact, as mentioned before, these polyunsaturated fats inhibit lipogenesis and stimulate fatty acid oxidation. The results obtained seems to be promising to improve NAFLD in children, however further studies need to be made amplifying sample size of patients treated and making homogenous trial design before to suggest their use in NAFLD treatment.

In clinical research, there is a widespread interest in seeking for new drug to be used both in children and adults with NAFLD. Although many drugs are being approved by the FDA to treat NAFLD in adult, no medication is currently available in pediatric patient with NAFLD.

NASPGHAN recommends bariatric surgery as treatment for adolescents (aged more than 12 years) with a BMI ≥35 kg/m and major comorbidities such as T2DM or NASH with advanced fibrosis, or BMI ≥40 kg/m^2^ with milder comorbidities (2).

Pending the development of more accurate biomarkers to non-invasively assess improvement in NAFLD, the decrease in ALT from baseline may be useful as a surrogate marker of the effectiveness of therapy, especially in the first year of treatment. The regression of fibrosis needs to be evaluated through liver biopsy after a longer period of treatment (> 2 years) (2).

## Conclusions

NAFLD is an emerging medical condition influencing pediatric patient’s health. The growing number of young people affected with obesity is leading to an increased occurrence of hepatic steatosis and its correlated comorbidities in pediatric patients with an alarming worldwide prevalence. It consists of a disease with a multifactorial etiopathogenesis. In fact, although environmental factors are widely proven to be associated with hepatic steatosis, it is getting stronger the evidence of a genetic background influencing the onset and progression of disease. IR and obesity are the main risk factors correlated to disease, but also age, sex, and ethnicity can play an essential role. Furthermore, NAFLD is part of the wide spectrum of metabolic alterations of MetS, ranging from impaired glucose tolerance to dyslipidemia and hypertension. The development and progression of the disease in childhood lead to an increased risk of long-term morbidity, especially for cardiovascular diseases. Therefore, therapeutic approaches aiming to lifestyle changes with weight loss and to modify the major risk factors need to be undertaken.

## Author Contributions

SS and CG drafted the manuscript. SS and ED’A collaborated in collecting data. CG revised the manuscript for important intellectual content. FC and AM provided critical feedback. All authors contributed to the article and approved the submitted version.

## Conflict of Interest

The authors declare that the research was conducted in the absence of any commercial or financial relationships that could be construed as a potential conflict of interest.
